# Breathlessness and incidence of COPD, cardiac events and all-cause mortality: A 44-year follow-up from middle age throughout life

**DOI:** 10.1371/journal.pone.0214083

**Published:** 2019-03-18

**Authors:** Jacob Sandberg, Gunnar Engström, Magnus Ekström

**Affiliations:** 1 Lund University, Faculty of Medicine, Department of Clinical Sciences Lund, Respiratory Medicine and Allergology, Lund, Sweden; 2 Dept of Clinical Sciences Malmö, Lund University, Malmö, Sweden; Leibniz Institute for Prevention Research and Epidemiology BIPS, GERMANY

## Abstract

**Background:**

Breathlessness is prevalent in the general population and may be associated with adverse health outcomes. This study aimed to evaluate the association of breathlessness with Chronic Obstructive Pulmonary Disease (COPD) events, cardiac events and all-cause mortality from middle-age throughout life.

**Methods:**

Breathlessness was measured in 699, 55-year old men residing in Malmö, Sweden using modified Medical Research Council (mMRC). COPD events (hospitalisation, death or diagnosis) cardiac events and all-cause mortality was assessed using The Swedish Causes of Death Register and Hospital Discharge Register. Data was analyzed using Cox- and competing risks (Fine-Gray) regression analysis.

**Results:**

695 (99%) of 699 participants died and four emigrated during follow up. Eighty-seven (12%) had mMRC = 1 and 19 (3%) had mMRC≥2. Breathlessness was associated with COPD events; adjusted Sub-Hazard Ratio 2.1 (95% CI, 1.2–3.6) for mMRC = 1 and 7.5 (2.6–21.7) for mMRC ≥ 2 but not associated with cardiac events when adjusting for competing events and confounding. Breathlessness was associated increased all- cause mortality (Hazard Ratios of 1.4 (1.1–1.7) (mMRC = 1) and 3.4 (2.1–5.6) (mMRC ≥ 2)).

**Conclusion:**

Breathlessness is associated with increased risk of COPD events and increase in all-cause mortality from age 55 until death.

## Introduction

Breathlessness is the subjective experience of breathing discomfort.[1] It has a high prevalence across a range of disorders such as chronic obstructive pulmonary disease (COPD) and chronic heart failure and is common among the general public.[2]

The modified Medical Research Council (mMRC) scale is a frequently utilized tool to measure breathlessness in both clinical settings and population studies.[1, 3, 4] It was developed in the 1950’s with the main purpose to categorize the functional impact and disability related to breathlessness and is currently recommended by international guidelines for categorizing the severity of COPD.[5]

Breathlessness is associated with mortality both due to cardiovascular diseases and COPD.[6–9] The presence of even mild breathlessness is a predictor of myocardial infarction, heart failure and death, also in people without known heart disease.[7, 8] Among people suffering of COPD, the intensity of breathlessness, measured with mMRC, predicts 5 year mortality better than spirometry values of forced expiratory volume in one second (FEV_1_).[10]

The knowledge is limited on the association between breathlessness and future COPD and cardiac events, and on the association with mortality throughout the life span and no previous studies has been conducted using competing events regression(Fine and Gray’s). Early detection of people with an increased risk is vital to ensure early treatment measures as well as to give correct information which might enhance willingness to make lifestyle changes or adhere to treatment.

This study aimed to evaluate the association of breathlessness with Chronic Obstructive Pulmonary Disease (COPD) events, cardiac events and all-cause mortality from middle-age throughout life using competing risks regression. Possible differences in associations according to different trajectories of breathlessness will also be explored.

## Materials and methods

### Study design and population

This study is based upon the “Men Born in 1914” cohort, which started with baseline examinations performed in 1968 in Malmö, Sweden. Invitation to participate went out to all men who were born in even-numbered months in 1914. Out of 809 eligible individuals, 703 (87%) participated in the baseline survey. Four participants were excluded for not answering the mMRC rating at baseline, leaving a total of 699 included participants in the present study. Those still alive and still living in Malmö were asked to be re-examined in 1982–83, and 407 of 482 individuals (81%) agreed. The 297 men who did not participate were either dead (n = 132), had moved away (n = 75) or chose not to participate (n = 90). Follow up lasted until the last participant died in 2013.

### Assessments

The mMRC was measured at baseline in 1968 and at follow-up in 1982–83. The mMRC is self-administered with categories 0, “Not troubled by breathlessness, except with strenuous exercise”, 1, “Troubled by shortness of breath when hurrying on the level or walking up a slight hill”, 2, “Breathless or has to stop for breath when walking at own pace on the level”, 3, " Stops for breath after walking about 100 yards (90m) or after a few minutes on the level” and lastly 4 “I am too breathless to leave the house or I am breathless when dressing”.[1, 3, 4, 11] Participants were divided into three groups according to the mMRC rating (mMRC = 0, mMRC = 1 and mMRC ≥ 2).

Spirometry testing, without prior bronchodilation, was available for 689 (98.6%) participants at baseline. Forced Expiratory Volume in one second (FEV_1_) and vital capacity (VC) was measured and used to calculate FEV_1_% of predicted using European reference values.[12] Airflow limitation at baseline was defined as having a FEV_1_/VC < 0.7.[13]

Body mass index (BMI) was calculated using body weight and height and categorized into underweight (≤18.5), normal weight (18.5–25), overweight (25–30) and obese (>30). Smoking status was categorized as “currently smoking”, “previously smoking” and “never smoking”. Hypertension was defined as a blood pressure above 140/90. Physical activity ranged from “regular hard physical activity”, “regular activity”, “some physical activity” and “almost inactive”. Details of the questionnaires and the assessments have been published elsewhere.[14, 15]

### Outcomes

Primary outcomes were COPD events, cardiac events and all-cause mortality. COPD events were defined as the first occurrence of a COPD-related hospitalisation, death or diagnosis. Hospitalisation were established from discharge summaries following hospital care, death from death certificates and diagnosis from out-patient registries from Swedish hospitals. International Classification of Diseases (ICD)-8 (1968–1986; codes 490–492), ICD-9 (1987–1997; codes 490–492 and 496) and ICD-10 (1997–2013; codes J40–J44) were used to establish the COPD event.

Cardiac events were defined as hospitalisation due to myocardial infarction (ICD-9 code 410 and ICD 10 code I21) or death due to ischemic heart disease (ICD 9 codes 410–414; ICD 10 codes I21-I25).

Secondary outcomes were *incident* COPD events using participants without airflow limitation (FEV_1_/VC <0.7) at baseline and *incident* cardiac events in people without previous myocardial infarction at baseline.

The trajectory of breathlessness was investigated using data from the follow up examination performed in 1982 and categorised into four groups: Continuous breathlessness (breathlessness present at both baseline and follow-up), remitting breathlessness (breathlessness at baseline but not in 1982) and incident breathlessness (asymptomatic at baseline but breathless in 1982). These groups were compared to the “never breathless” participants (reference category).

The Swedish inpatient registry has been found to be of acceptable validity and good specificity for COPD-disease in epidemiological research and has been active in the south of Sweden for the whole study period.[16].

### Statistical analyses

Baseline patient characteristics were expressed as frequencies and percentages and compared between the breathlessness groups using Pearson’s Chi-Square for categorical variables and one-way ANOVA for continuous.

COPD events, cardiac events and all-cause mortality were visualised by breathlessness group using Cumulative incidence curves and Kaplan-Meier plots (tested using log rank test). The associations between breathlessness, COPD events and cardiac events were analysed using competing risks regression according to Fine and Gray’s proportional subhazards model with non-COPD or non-CE deaths as competing events.[17] Association between breathlessness and all-cause mortality were analysed using Cox proportional hazards regression. The time from baseline assessment to the first of COPD event, cardiac event, death or emigration (n = 4) from Sweden was used to assess the rate of COPD events, cardiac events and all-cause mortality over the total 44 years of follow-up.

All results were adjusted for available potential confounders including smoking, diabetes, BMI, level of physical activity, dyslipidaemia, height, and hypertension. FEV_1_%predicted was chosen to adjust for lung function impairment as has been performed in previous similar studies. [6, 9] The COPD events were additionally adjusted for having airflow limitation at baseline (FEV_1_/VC<0.7) and the cardiac events for having had a previous myocardial infarction (MI) before baseline.

*Incident* COPD events were analysed among only the participants without airflow limitation (FEV_1_/VC<0.7) at baseline, and *incident* cardiac events were analysed in only the participants without previous MI at baseline. Because of the lower number of breathless individuals in theses analyses, the breathlessness groups had to be merged to a joint category of mMRC ≥ 1. The same hade to be done when examining the associations between the trajectory of breathlessness (continuous, remitting, incident and never) and COPD events, cardiac events and all-cause mortality using competing risks regression with adjustments for confounding effects as described above.

Statistical significance was defined as two-sided p-value < 0.05. Statistical analyses were conducted using the software package Stata, version 14.2 (StataCorp LP; College Station, TX).

### Ethical considerations

All participants were informed and gave verbal consent to participate in the study which were in accordance with research regulations and laws at the period of the study. The study was approved by the Regional Ethical Review Board in Lund, Sweden (DNr 1982–111 and 2013–443).

## Results

### Patient characteristics

Baseline characteristics of the 699 included men are shown in Table 1. A majority (62%) where current smokers, approximately 40% were hypertensive, 2% diabetic and 43% were overweight or obese. 106 (15%) participants had any grade of breathlessness on the mMRC scale, nine (1%) had had a myocardial infarction, and 144 (21%) participants had airflow limitation (FEV_1_/VC < 0.7) at baseline. (Table 1)

**Table 1:** Baseline characteristics of 699 participants per modified Medical Research Council (mMRC) breathlessness score at age 55.

Factor	mMRC = 0	mMRC = 1	mMRC≥2	p-value
Subjects (n)	593	87	19	
Body Mass Index (kg/m^2^)				<0.001
≤18.5	13 (2.2%)	1 (1.2%)	0 (0.0%)	
18.5–25	327 (55.4%)	43 (50.0%)	7 (36.8%)	
25–30	233 (39.5%)	34 (39.5%)	8 (42.1%)	
>30	17 (2.9%)	8 (9.3%)	4 (21.1%)	
Smoking				0.14
Never	97 (16.4%)	7 (8.0%)	3 (15.8%)	
Former	138 (23.3%)	16 (18.4%)	3 (15.8%)	
Current	358 (60.4%)	64 (73.6%)	13 (68.4%)	
Hypertension	237 (40.0%)	36 (41.4%)	10 (52.6%)	0.53
Diabetes	11 (1.9%)	3 (3.5%)	1 (5.6%)	0.38
Cholesterol (mmol/L), mean (SD)	6.4 (1.1)	6.2 (1.2)	6.2 (0.9)	0.45
Physical activity				0.013
Regular hard	14 (2.4%)	0 (0.0%)	0 (0.0%)	
Regular	82 (14.0%)	7 (8.1%)	0 (0.0%)	
Some	333 (57.0%)	43 (50.0%)	10 (52.6%)	
Inactive	155 (26.5%)	36 (41.9%)	9 (47.4%)	
FEV_1_% of predicted, mean % (SD)	90 (40)	90 (20)	80 (20)	0.13
FEV_1_/VC mean (SD)	0.77 (0.08)	0.73 (0.1)	0.70 (0.1)	<0.001
Airflow obstruction at baseline (FEV_1_/VC <0.7), n (%)	103 (17.7)	31 (36.0)	10 (52.6)	<0.001
Cardiac event before baseline, n (%)	5 (0.8)	3 (3.4)	1 (5.3)	0.039

Data presented as frequency (%) if not otherwise stated. Categorical data compared using Pearson’s Chi-square. Continuous data compared using one-way ANOVA. FEV_1_ = forced expiratory volume during 1 second; VC = vital capacity; SD = Standard deviation

Thirteen percent (n = 89) experienced a COPD event throughout the follow-up time, mainly through hospitalisation (n = 81), but for a few through diagnosis at out-clinic visits (n = 2), or from death certificates (n = 6, of which 5 were confirmed by autopsy). A cardiac event occurred in 276 participants (39%). (Table 2)

**Table 2:** Association between breathlessness and chronic obstructive pulmonary disease (COPD) events, cardiac events and all-cause mortality.

	mMRC = 0	mMRC = 1	mMRC ≥2
Subjects (n)	593	87	19
**COPD events (Fig 1)**			
Events, n (n per 1000 person-years)	59 (4.3)	21 (13.1)	9 (42.6)
Crude SHR (95% CI)	1.00	2.7 (1.6–4.4) ¤	7.2 (3.3–16.0) ¤
Adjusted SHR (95% CI) * #	1.00	2.1 (1.2–3.6) §	7.5 (2.6–21.7) ¤
**Cardiac events (Fig 2)**			
Events, n (n per 1000 person-years)	233 (17.9)	35 (21.2)	8 (31.7)
Crude SHR (95% CI)	1.00	1.1 (0.7–1.5)	1.2 (0.6–2.7)
Adjusted SHR (95% CI) * ^	1.00	0.9 (0.6–1.4)	0.6 (0.2.-1.7)
**All-cause mortality (Fig 3)**			
Deaths, n (n per 1000 person-years)	589 (42.5)	87 (50.5)	19 (73.1)
Crude HR (95% CI)	1.00	1.5 (1.2–1.9) ¤	3.6 (2.2–5.7) ¤
Adjusted HR (95% CI) /	1.00	1.4 (1.1–1.7) §	3.4 (2.1–5.6) ¤

mMRC = modified Medical Research Council, SHR = Sub Hazard Ratio, HR = Hazard Ratio

/ Adjusted for smoking status (three groups: never, former- and current smokers), FEV1%predicted, body mass index, height and physical activity

* additionally adjusted for hypertension, dyslipidaemia and diabetes

# additionally adjusted for airflow limitation at baseline

^additionally adjusted for the presence of cardiac event before baseline

¤ p<0.001

§ p<0.05

A total of 695 out of 699 participants (99%) died during the follow up, and the remaining four were lost to followup due to emigration. (Table 2)

**Fig 1:**
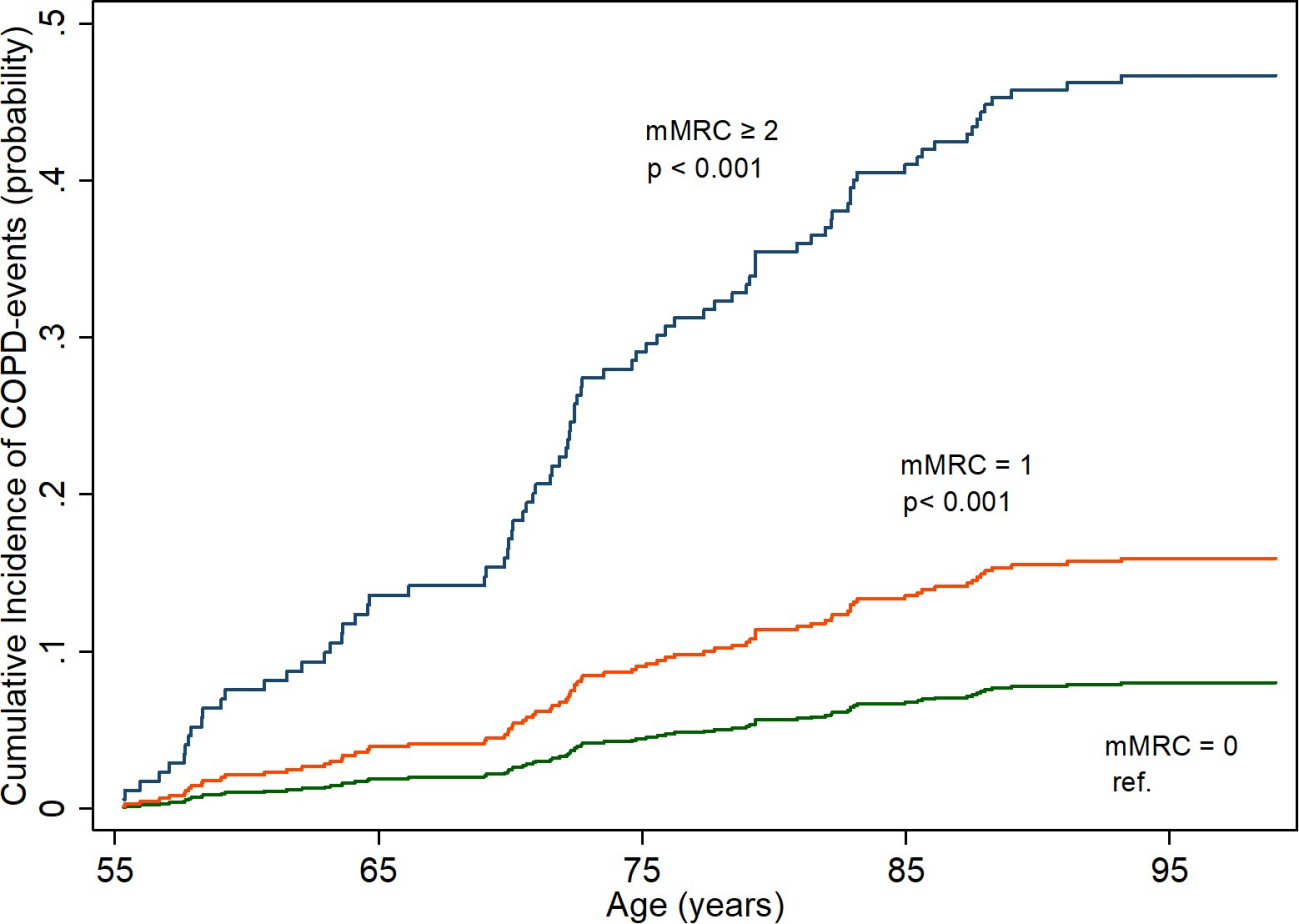
Risk of chronic obstructive pulmonary disease (COPD) events (hospitalisation, out-clinic diagnosis or diagnosis from death-certificate) per modified Medical Research Council (mMRC) grade from age 55 throughout life. Calculated using competing risks regression.

COPD and cardiac event rates, hazard ratios (HR) and subhazard ratios (SHR) per mMRC grade at age 55 are presented in Table 2. Compared to participants without breathlessness, participants with breathlessness level of mMRC = 1 had an increased risk of COPD event throughout life as shown in Fig 1, the adjusted SHR was 2.1 (95% CI, 1.2–3.6). For the individuals with more breathlessness (mMRC ≥ 2) the risk increased and the adjusted SHR was 7.5 (95% CI, 2.6–21.7).

**Fig 2:**
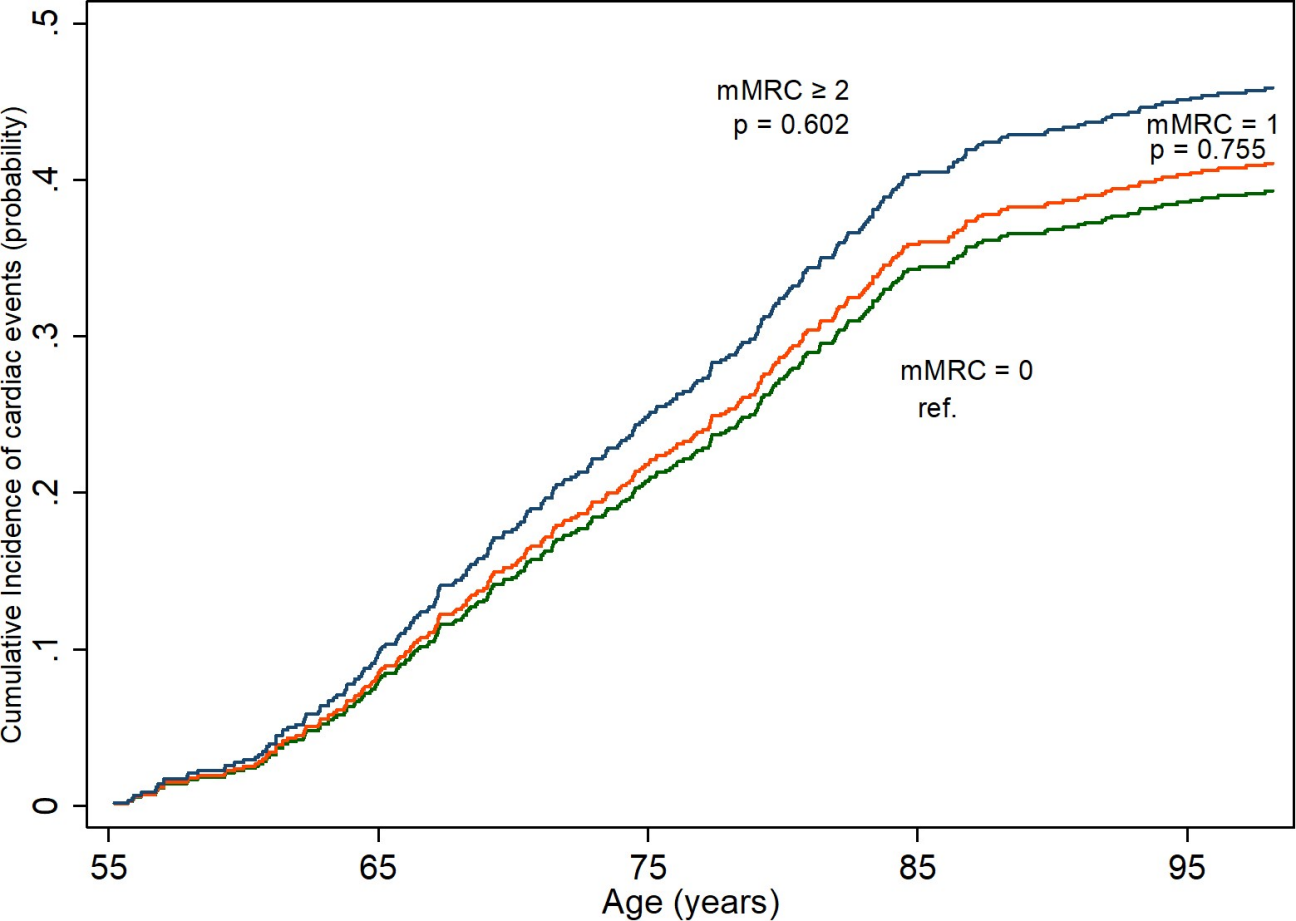
Risk of cardiac events (hospitalisation or death certificates) per modified Medical Research Council (mMRC) grade from age 55 throughout life. Calculated using competing risk regression.

Breathlessness was not significantly associated with cardiac events when adjusted and calculated with competing risks analyses. Adjusted SHR was 0.9 (95%CI 0.8–1.7) for mMRC = 1 and 0.6(95%CI 0.2–1.7) for mMRC ≥ 2 (Table 2, Fig 2) When using cox-regression (not accounting for competing events) a trend for higher risk, significant for the higher grade of breathlessness was shown with crude HR of 2.7 (95% CI, 1.3–5.6) and adjusted HR of 2.0 (95% CI, 0.9–4.4).

**Fig 3:**
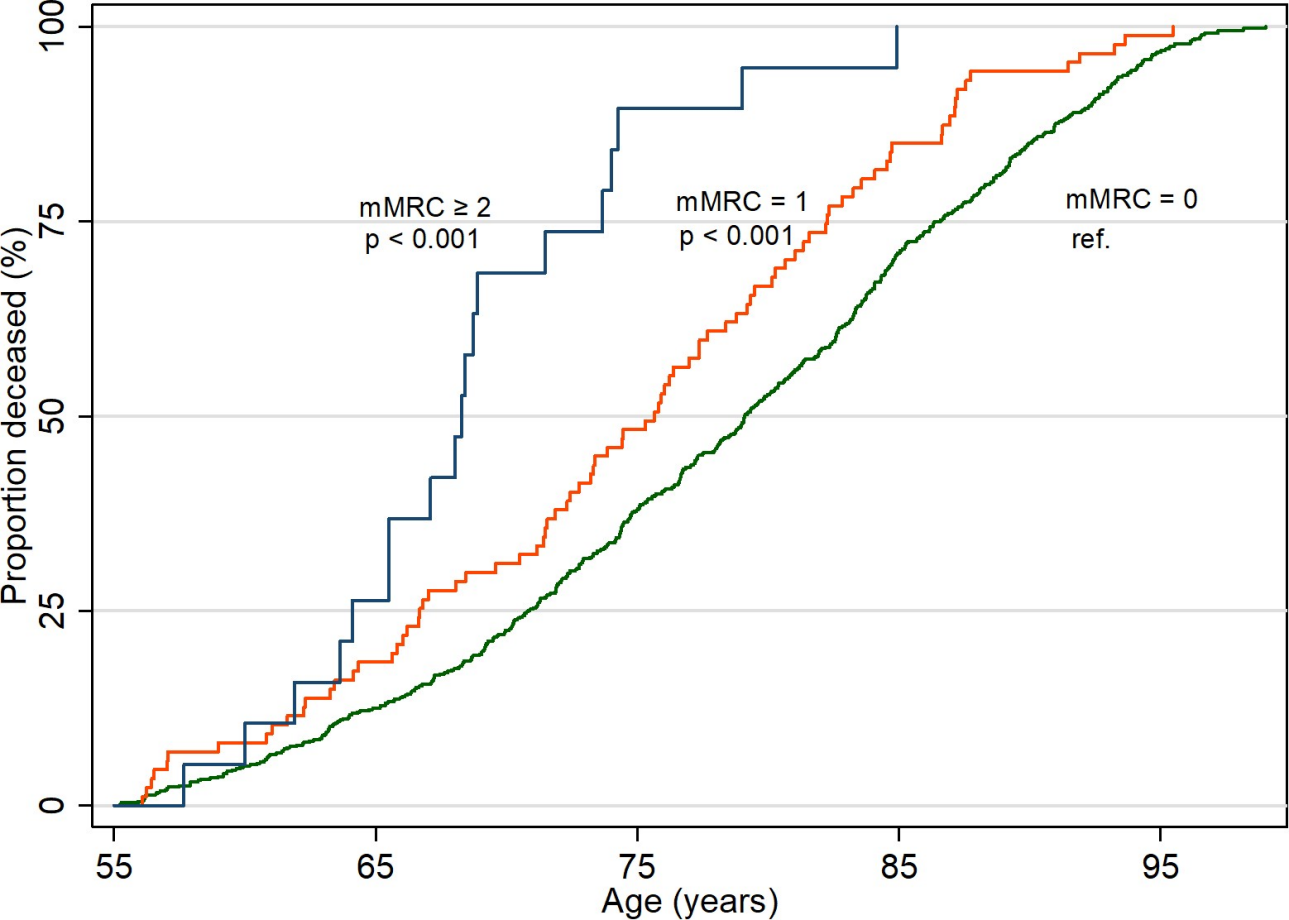
All-cause mortality per modified Medical Research Council (mMRC) grade from age 55 and throughout life. P-values were calculated using log rank tests.

Higher mMRC grades were significantly associated with a higher all-cause mortality with a HR of 1.4 (95% CI, 1.1–1.7)) for mMRC = 1 and HR 3.4 (95% CI, 2.1–5.6) for mMRC ≥ 2. (Table 2, Fig 3)

When all 144 individuals with airflow limitation (FEV_1_/VC < 0.7) at baseline were removed from analyses, the crude SHR for incident COPD event was 2.4 (95% CI, 1.2–5.0) and 2.2 (95% CI, 1.0–4.8) when adjusted. (Table 3) No association was shown between breathlessness and incident cardiac events (participants with myocardial infarction prior to baseline removed).

**Table 3:** Association between breathlessness and incident chronic obstructive pulmonary disease (COPD) events and incident cardiac events. Analyses were performed in participants with normal lung function or no previous cardiac event at baseline, respectively.

	mMRC = 0	mMRC≥1
**Incident COPD events**		
Subjects (n)	490	65
Events, n (n per 1000 person-years)	33 (2.8)	10 (8.0)
Crude SHR (95%CI)	1.00	2.4 (1.2–5.0) ¤
Adjusted SHR (95%CI) *	1.00	2.1 (1.0–4.6) §
**Incident cardiac events**		
Subjects (n)	587	102
Events, n (n per 1000 person-years)	228 (17)	40 (21)
Crude SHR (95%CI)	1.00	1.0 (0.7–1.5)
Adjusted SHR (95%CI) *	1.00	0.9 (0.6-1-3)

mMRC = modified Medical Research Council, SHR = Sub Hazard Ratio

*Adjusted for smoking status (three groups: never, former- and current smokers), FEV1%predicted, diabetes, body mass index, height, hypertension, dyslipidaemia and physical activity

¤ p = 0.014

§ p = 0.056

Continuous breathlessness (n = 37) was associated with a higher risk of COPD events (SHR 3.2 (95% CI, 1.5–6.7)) and a higher all-cause mortality, HR 2.2 (95%CI,1.5–3.2). Incident breathlessness (n = 80), was associated with COPD events, however not significant after adjustments (SHR 1.7 (0.8–3.3)) and with a higher all-cause mortality, HR 1.5 (95% CI, 1.1–1.9). The participants with remitting symptoms were very few (n = 9) and had similar risks to the not breathless group. (Table 4)

**Table 4:** Incidences and associations between breathlessness (mMRC>1) and chronic obstructive pulmonary disease (COPD) events, cardiac events and all-cause mortality by trajectory of breathlessness using data from baseline and follow-up in 1982–83.

	Never breathlessness	Continuous breathlessness	Incident breathlessness	Remitting breathlessness
Subjects (n)	272	37	80	97
**COPD events**				
Events n (n per 1000 person-years)	25 (3.2)	14 (18.0)	14 (7.3)	2 (8.2)
Crude SHR (95% CI)	1.00	5.3 (2.7–10.2) ¤	2.1 (1.1–4.1) §	2.8 (0.6–12.4)
Adjusted SHR (95% CI) * #	1.00	3.2 (1.5–6.7) §	1.7 (0.8–3.3)	2.5 (0.5–12.7)
**Cardiac events**				
Events, n (n per 1000 person-years)	102 (13.9)	17 (20.5)	37 (20.6)	2 (8)
Crude SHR (95%CI)	1.00	1.4 (0.8–2.3)	1.4 (1.0–2.3)	0.5 (0.1–2.3)
Adjusted SHR (95% CI) * ^	1.00	1.2 (0.7–2.1)	1.4 (0.9–2.2)	0.5 (0.1–2.3)
**All-cause mortality**				
Deaths n (n per 1000-person years)	272 (35.3)	37 (42.9)	80 (39.5)	9 (34.2)
Unadjusted HR (95% CI)	1.00	2.3 (1.6–3.3) ¤	1.4 (1.1–1.9) §	1.08 (0.6–2.1)
Adjusted HR (95% CI) /	1.00	2.2 (1.5–3.1) ¤	1.5 (1.1–1.9) §	1.0 (0.5–2.0)

mMRC = modified Medical Research Council, SHR = Sub Hazard Ratio, HR = Hazard Ratio

/ Adjusted for smoking status (three groups: never, former- and current smokers), FEV1%predicted, body mass index, height and physical activity.

*Additionally adjusted for diabetes, hypertension and dyslipidaemia

# additionally adjusted for airflow limitation at baseline

^additionally adjusted for the presence of cardiac event before baseline

¤ p < 0.001

§ p < 0.05

## Discussion

### Main findings

This study shows that being breathless in the middle age is highly associated with poorer health outcomes throughout life in terms of markedly increased risk of COPD events (hospitalisations, out-patient diagnosis or COPD-related death) and earlier death overall. Interestingly, this study shows that even low-grade breathlessness (mMRC = 1; “when hurrying or walking up a steep hill”) is associated with higher rate of COPD events and overall mortality even after adjustments for potential confounders. When individuals with airflow limitation (FEV_1_/VC<0.7) at baseline were removed, a risk increase for incident COPD-diagnosis remained although not significant when fully adjusted. This could be due to lack of power. The finding indicates that low-grade breathlessness has a prognostic value for future incident COPD and death and could be a risk factor for COPD even when lung function is normal. Breathlessness seems to be an independent risk factor for both COPD-events and all-cause mortality as the findings are all adjusted for lung function as well as other risk factors.

Associations between breathlessness and future cardiac events were weak and not significant. A significant trend for higher risks of cardiac events and mortality with increasing breathlessness was found when using Cox regression but when using competing risks analysis, the association disappeared. This was further reinforced when adjusting for confounding effects from smoking status FEV1%predicted, diabetes, body mass index, height, hypertension, dyslipidaemia, physical activity and having had a myocardial infarction already at baseline. These findings are interesting as they contradict previous studies which has shown an association between breathlessness and myocardial infarction.[7] Our findings may indicate that the increased risk shown previously might be mediated in large by respiratory complications and death and when accounting for these by using competing risks regression the risk increase does not remain. It might also be possible that there are missing cases of sudden cardiac death which may not have been categorised as myocardial infarction, however almost all causes of deaths were established from autopsy which should reduce this weakness.

The results in this study strengthens findings from previous studies, [6–8, 18] with larger study populations but shorter follow-up times and mostly focusing on mortality and none specifically on COPD events. We also found that continuous, chronic breathlessness is the most associated with poor health outcomes and that participants with remitting breathlessness returned to the risk of the normal population. This is consistent with the only other report, to our knowledge, on this topic.[6]

### Strengths and limitations

This is a prospective longitudinal study with a very long follow up of middle aged individuals followed throughout life, regarding breathlessness and its relationship with COPD events, cardiac events and overall mortality. A strength compared with previous studies is that this study accounted for competing risks which has not been done before. The outcome data is very reliable as the registries have been active with near complete coverage for the whole study period, Validation studies has been performed [16] and most fatal events are based upon autopsy results (5 out of 6 COPD deaths). Limitations of this study include that only males were studied. However, previous studies which included both men and women showed similar associations between genders for the association between breathlessness and all-cause mortality [18, 19] It is also possible that milder COPD events which never required hospital admission have been missed, since almost all the COPD-incidence is based on hospital discharge summaries, and diagnoses from primary care facilities were not available.

The long follow-up gives a lot of strengths to this study but at the same time adds a risk of changes within the baseline characteristics over time which would affect risks. Many of the participants were smokers at baseline but had quit smoking at follow-up which could lead to an underestimation of the associations between breathlessness and mortality as that risk factor were adjusted for but actually had disappeared.

Limitations also include that we were not able to adjust for socioeconomic factors as data did not exist as well as the low number of participants with remitting breathlessness which makes estimates less reliable.

### Implications

This study further showcases the need for the clinician to take prompt interest in patients with even a low grade self-reported breathlessness with or without reduced lung function. Patients presenting with chronic breathlessness has a wide variety of underlying diagnoses, but the majority is respiratory.[20, 21] Our study further highlights the need to establish a diagnosis in these patients and ensure intervention such as smoking cessation, increased physical activity, control of other risk factors and medication. Future research should focus on how to better and earlier identify patients with breathlessness as well as how to intervene in the most effective way when these individuals presents in, most commonly, a primary care setting. There is also a need for further research with larger populations on the associations between breathlessness and cardiac events, as our study showed no association when accounting for competing events.

## Conclusion

In conclusion, this study shows that presence of breathlessness at 55 years of age is associated with an increased risk of COPD events and increase in all-cause mortality throughout life.
